# Endoscopic endonasal pituitary surgery: a rhinologist point of view

**Published:** 2012-11

**Authors:** Maryam Jalessi, Guive Sharifi

**Affiliations:** ^*a*^Endoscopic Pituitary and Skull Base Surgery Center, ENT-Head & Neck Research Center and Department, Hazrat Rasoul Akram Hospital, Tehran University of Medical Sciences, Tehran, Iran.; ^*b*^Neurosurgery Department, Loghman Hakim Hospital, Shaheed Beheshti University of Medical Sciences, Tehran, Iran.

## Abstract

Endoscopic endonasal approaches are increasingly becoming the state of art in transsphenoidal pituitary surgery. The goal of this study is first to briefly introduce nasal and sphenoidal phases of the approach along with reconstruction phase. Then, the key points of radiologic studies of various parts of the surgery with images and movies are discussed. Difficult cases from 350 surgeries will be showed and technical modifications in each phase according to later cases were noticed. Furthermore, the disadvantages and pitfalls are highlighted illustratively.

**Keywords:**

Endoscopic, Endonasal, Pituitary


					Volume 4, Suppl. 1 Nov 2012
Publisher: Kermanshah University of Medical Sciences
URL: http://www.jivresearch.org
Copyright:
Congress of Iranian Neurosurgeons, 14-16 November 2012, Kermanshah, Iran
Paper No. 21
Endoscopic endonasal pituitary surgery: a rhinologist point of
view
Maryam Jalessi a,*
, Guive Sharifi b
a
Endoscopic Pituitary and Skull Base Surgery Center, ENT-Head & Neck Research Center and Department, Hazrat Rasoul
Akram Hospital, Tehran University of Medical Sciences, Tehran, Iran.
b
Neurosurgery Department, Loghman Hakim Hospital, Shaheed Beheshti University of Medical Sciences, Tehran, Iran.
Abstract:
Endoscopic endonasal approaches are increasingly becoming the state of art in transsphenoidal pituitary surgery.
The goal of this study is first to briefly introduce nasal and sphenoidal phases of the approach along with
reconstruction phase. Then, the key points of radiologic studies of various parts of the surgery with images and
movies are discussed. Difficult cases from 350 surgeries will be showed and technical modifications in each phase
according to later cases were noticed. Furthermore, the disadvantages and pitfalls are highlighted illustratively.
Keywords:
Endoscopic, Endonasal, Pituitary
* Corresponding Author at:
Maryam Jalessi: Endoscopic Pituitary and Skull Base Surgery Center, ENT-Head & Neck Research Center and Department, Hazrat Rasoul Akram
Hospital, Tehran University of Medical Sciences, Tehran, Iran, Tel: 09123000332, Email: mi.shafizad@gmai.com, (Jalesi M.).

				

